# A review on the relationship between anti-mullerian hormone and fertility in treating young breast cancer patients

**DOI:** 10.1186/s12905-021-01420-3

**Published:** 2021-08-10

**Authors:** Yixuan Song, Hong Liu

**Affiliations:** The Second Surgical Department of Breast Cancer, Tianjin Medical University Cancer Institute and Hospital, National Clinical Research Center for Cancer, Key Laboratory of Cancer Prevention and Therapy, Tianjin’s Clinical Research Center for Cancer, Tianjin, China

**Keywords:** Young breast cancer, Fertility, AMH, Metabolism, BRCA gene

## Abstract

Despite the fact that the long-term survival rate of breast cancer patients had been significantly improved owing to the systemic breast cancer therapies, there are still some side effects such as amenorrhea and fertility retention to be resolved, leaving it an important thing to understand the possible side effects on fertility and fertility preservation strategies while undergoing breast cancer treatment, due to the fact that most young patients hope to become pregnant and have children after breast cancer treatment. With anti-müllerian hormone (AMH) being the most sensitive marker for predicting ovarian function in young premenopausal women with breast cancer, this review is aimed to provide the additional guidance for clinical application of AMH by exploring the impacts of AMH on the fertility of young breast cancer patients, the relationship between AMH and metabolism, and the relationship between BRAC gene mutation and fertility protection strategies.

## Background

In recent years, breast cancer is one of the most common malignancies among women, with the increasing occurrence [1]. For premenopausal breast cancer patients, included are such treatment strategies as surgery, cytotoxic chemotherapy, endocrine therapy, radiation therapy, and targeted therapy^2^, which, despite the improved survival rates, may have side effects such as early menopause and fertility disorders [2]. Based on a meta-analysis, the pregnancy probability of women receiving systemic treatment after surgery is about 14%, and the pregnancy rate of the survivors after receiving breast cancer treatment is on average 40% lower compared with general female [3], leaving many female patients concerned about infertility in the future and early menopausal symptoms [4]. The fertility preservation can be assessed with ovarian reserve biomarkers such as anti-müllerian hormone (AMH), inhibin B levels, antral follicle count (AFC), early follicular phase follicle stimulating hormone (FSH), and estradiol [5]. The recruitment of primitive follicles and the effect of FSH on follicles during growth can be inhibited by AMH, a glycoprotein hormone in the transforming growth factor-beta (TGF-β) family [6], which is supported by Ruddy et al. that with aging, the risks of amenorrhea at 12 months and 18 months after chemotherapy increase by 20% and 18% respectively, while with each 1 ng/mL increase in AMH, the chance of amenorrhea at the 18th month of chemotherapy decreases by 59%[7]. Nowadays, AMH has been shown, in young premenopausal women with breast cancer, to be the most sensitive marker for predicting ovarian function recovery and premature ovarian insufficiency [8]. The purpose of this review is to discuss whether there is a correlation between AMH and fertility in young patients with breast cancer, thus guiding the selection of fertility protection strategies, with the association between metabolism and AMH examined to assess the impact of metabolism on fertility.

## Protective mechanism of AMH

Produced by growing follicular granulosa cells is AMH, known as Müllerian inhibiting substance (MIS), which can negatively regulate primordial follicles' activation to prevent over-recruitment. It was revealed that, by the presence of more primordial follicles in MIS-treated mice with chemotherapy drugs than that in control mice, the activation of primordial follicles could be blocked by MIS, which was shown to be possibly effective in protecting primary ovarian function throughout chemotherapy [9], while primordial follicles (PMFs), which relate to the reverse of follicular ovarian, could be activated by phosphoinositide 3-kinase (PI3K) signaling pathway and be inhibited by AMH. PMFs are activated by Cyclophosphamide (Cy), an alkylating agent commonly used to treat breast cancer, via up-regulating the PI3K-PTEN-AKT pathway in the ovary, resulting in premature ovarian failure, the gonadal toxicity contradicting the traditional theory that traditional chemotherapy's direct effect on oocyte DNA in PMF makes these resting follicles apoptotic [10]. As shown in the model of AMH administration to a Cy-treated pubertal mice, PMF reduction could be restrained by inhibiting FOXO3A phosphorylation, and AMH administration can induce ovarian autophagy that is inhibited by Cy-activated PI3K pathway, of which the signaling pathway was not activated by AMH, implying that the mechanism of AMH-induced ovarian autophagy has a correlation with FOXO3A, pointing to a hypothesis that AMH is closely related to autophagy which can protect PMF reserves and limit the Cy-induced follicular consumption, leading to a consequence that AMH with chemotherapy combination might be a promising strategy of protecting the fertility of young breast cancer patients [11].

FOXO3A, transcription factor that can regulate autophagy, is essential for maintaining gene expression programs, with PI3K-PTEN-AKT-FOXO3A pathway shown to play a key role in primordial follicle activation and the phosphorylation of FOXO3A in the ovaries of AMH-treated mice significantly reduced, according to the studies on the PI3K signaling pathway. Expressed in the primordial follicles nucleus, FOXO3A is essential for maintaining PMF dormancy [12], for example, the activation of full follicles in one FOXO3A-deficient mouse resulted in oocyte death and insufficient early ovarian reserve [13]. In addition, the activation of primordial follicles was generated by phosphorylated FOXO3A via promoting protein transport out of the nucleus [14], leading to the result that AMH could keep FOXO3A located in the PMF nucleus, thus preventing PMF activation in chemotherapy by avoiding cytoplasmic transfer of FOXO3A [11].

Regulating the development of early follicular and ovarian follicles appears to be crucial [15], in that AMH not only inhibits the growth of primitive follicular by suppressing the functions of such stimulating factors as fibroblast growth factor (bFGF), Stem cell factor (SCF), a granular growth factor transducing through the phosphoinositide 3-kinase (PI3K) pathway by binding to oocyte c-Kit receptors, or keratinocyte growth factor (KGF) [16], but also, with AMH acting as a follicular autocrine/paracrine factor to control SCF expression through the cAMP/PKA pathway, which decreased the expression of SCF mRNA and protein in human granulosa cells [17], but also inhibits SCF expression in human granules by phosphorylating cAMP-response element-binding protein (CREB) through the cAMP/PKA signaling pathway, thus providing a better understanding of how the AMH molecular pathway suppresses follicular development, which will aid in the development of new therapies [18].

## AMH and chemotherapy

Among breast cancer survivors, chemotherapy, while its correlation with ovarian reserve markers is still very controversial [19], is identified to be related to the risk of amenorrhea, known as chemotherapy-induced amenorrhea (CIA), which can lead to premature ovarian failure by damaging ovarian reserve [20]. Based on a prospective cohort study of 239 women with breast cancer of childbearing age (follow up for 6 months), high AMH, compared to normal and low-basic AMH, led to a lower prevalence of chemotherapy-related amenorrhea (CRA), indicating that a high level of AMH residue after chemotherapy has certain protective effect on ovarian function, but it still needs further discussion [21].

### Evaluation of ovarian function after chemotherapy

Chemotherapy can lead to primary ovarian insufficiency due to follicle toxicity and indirect loss of primordial follicles through over-recruitment [22]. D’ Avila et al. found that serum AMH was significantly lower in patients after chemotherapy than before treatment, implying that serum AMH might be a helpful biochemical marker for predicting the degree of ovarian reserve impairment and the occurrence of amenorrhea [23]. The ASTRRA trial indicated that the accuracy of assessing menstrual recovery with age, estradiol, and AMH was 38.3%, 23.3%, and 86.7%, respectively, indicating that the AMH levels after chemotherapy were a comparatively accurate marker of ovarian function recovery [24]. Reduced AMH levels after chemotherapy were shown in a trial on 170 premenopausal breast cancer patients aged ≤ 40 years who received taxane-anthracycline-based chemotherapy, indicating an increasing rate of ovarian damage in patients [25], with AMH level diminishing to < 0.1 ng/ml (range < 0.1–0.21 ng/ml) in most patients (98.6%) at four weeks after chemotherapy [25]. An AMH testing showed that 73.3% (n = 101) of women did not recover ovarian function 2 years after chemotherapy (AMH < 0.1 ng/ml, range < 0.1–3.9 ng/ml) [25].

### Prediction of ovarian function before chemotherapy

Long-term ovarian function after chemotherapy can be predicted by the AMH level measured before chemotherapy, which could, in clinical practices, be conducive to predicting future fertility risks associated with chemotherapy [26]. According to a report by D'Avila et al., baseline AMH below 1.87 ng/ml could be used as an indicator of the risk of amenorrhea in women with breast cancer after the treatment with chemotherapy, and since amenorrhea indicates permanent impairment of ovaries, women of childbearing age with a baseline AMH below 3.32 ng/ml must be provided with various fertility preservation strategies against the possible impaired fertility after chemotherapy [27]. Additionally, serum AMH levels above 2 ng/mL before chemotherapy was shown by Dillon et al. to be capable of predicting better ovarian function recovery after chemotherapy [28]. And with the AMH level before chemotherapy found by a recent study to be related to 2-year amenorrhea in premenopausal early breast cancer patients with positive hormone receptor, 2-year amenorrhea could be precisely predicted with the combination of AMH levels and FSH levels before therapy, as well as age [29].

## AMH and endocrine therapy

Approximately 60% of premenopausal breast cancer patients will receive endocrine therapy alone or in combination with chemotherapy, using ovarian suppression or Tamoxifen clarified to be capable of enlarging chemotherapy toxicity as two possible options for endocrine therapy, depending on the patient and tumor [30], which was discovered by Jung et al. that breast cancer patients risk of chemotherapy-induced amenorrhea increased from 48 to 63.6% (P = 0.015) [31] with the use of Tamoxifen in chemotherapy and shown by a Cohort Study of young breast cancer women (≤ 40 years old) that that the incidence of chemical-induced amenorrhea was 15% for all patients, 13% for patients undergoing chemotherapy alone and 17% for patients receiving Tamoxifen after chemotherapy [32]. Nevertheless, another study showed that even though the fertility of Tamoxifen-treated patients with breast cancer was lower than that of those who did not receive treatment, there was a higher AMH level in Tamoxifen-treated women, suggesting no reduction in ovarian reserve (OR) [33]. Meanwhile, another study showed higher AMH in patients receiving the combination treatment of chemotherapy with Tamoxifen, with AMH levels of patients undergoing systemic cytotoxic treatment followed by endocrine therapy recovering quickly from 3 to 6 months [34]. Furthermore, despite limited evidence, the risk of fetal abnormalities (3.9% vs. 12.6%) in the general population was suggested to have increased, on the basis of a recent study, as the result of taking Tamoxifen during pregnancy [35].

## AMH and Trastuzumab therapy

Trastuzumab (T) is a human epidermal growth factor receptor-2 (HER2) monoclonal antibody that, when paired with adjuvant chemotherapy, can reduce the recurrence rate of HER2-positive early breast cancer by 50% [36], of which the administration in combination with 1-year adjuvant Trastuzumab to the HER2-positive breast cancer women was shown, by a protocol-specified analysis on 3222 HER2-positive breast cancer patients, to have dramatically increased disease-free survival [37]. NeoSphere trial indicates that neoadjuvant Pertuzumab in combination with Trastuzumab and Docetaxel can improve 5-year progression-free and disease-free survival in women with breast cancer [38].

The first study investigating amenorrhea rates with the combination treatment of Paclitaxel and Trastuzumab (APT Trial) illustrated that the rate of amenorrhea in premenopausal women treated with Paclitaxel and Trastuzumab, about 28%, was lower than that of the patients treated with standard adjuvant therapy [39]. The gonadal toxicity was suggested to be reduced by a systemic therapy in combination with Trastuzumab, based on a small prospective observational study involving 38 premenopausal breast cancer women who were proposed for adjuvant chemotherapy [8]. Patients receiving chemotherapy followed by Trastuzumab had a lower ovarian blood flow reduction than those receiving chemotherapy alone, according to Ben-Aharon et al. Besides, the use of Trastuzumab was shown, by a cross-sectional study, to be associated with elevated AMH in breast cancer survivors with normal menstruation [40], which was confirmed by a recent prospective observational study on premenopausal women with breast cancer who had undergone chemotherapy that patients receiving Trastuzumab had higher levels of AMH than those who did not [8].

## AMH and BRCA gene

BRCA genes are essential DNA double-strand break (DSB) repair, while the mutations in the genes can lead to a predisposition to cancers, including breast cancer and ovarian cancer [41], with the risk of breast cancer and contralateral breast cancer both increased by BRCA1 and BRCA2 mutations. According to a study, carriers of BRCA1 and BRCA2 combination, BRCA1, and BRCA2 mutations have a lifetime breast cancer risk of 70%, 24%, and 13%, respectively [42]. Observational studies have shown that BRCA1 mutation carriers giving birth at aged 30 years or older might have a lower risk of breast cancer, with a higher risk of breast cancer of a BRCA2 mutation carrier's first pregnancy happening before the age of 30 serving as a contrast [43].

A prospective study showed that women with BRCA mutations had significantly lower ovarian reserve than those without or at low risk [44]. Oktay, K. discovered that, of 126 breast cancer women, the BRCA mutation carriers had fewer ovarian oocytes than the non-carriers after stimulating their ovaries with Letrozole and gonadotropinss, an women with a positive BRCA1 mutation produced fewer ovarian oocytes and were 38.3 times more likely to have a low response than women with a positive BRCA2 mutation [45]. Contrary to these reports, no significant reduction in ovarian reserve or response to ovarian stimulation was found in a five-year retrospective cohort study on the in vitro fertilization (IVF) between BRCA mutations carriers and non-carriers, indicating no significant differences in the IVF cycles or in the number of oocytes [46, 47], which requires further studies due to the small sample size.

Laboratory studies show that BRCA Mutations decrease the number of primordial follicles and accelerate the accumulation of DNA double-strand breaks in oocytes, that women with BRCA1 mutations typically have a less ovarian reserve and earlier menopause, that the ATM-mediated DNA repair pathway associated with BRCA was shown by the available data to be a regulator of oocyte aging that might be detrimental to oocyte health, which could open up the possibility of developing targeted therapies to reverse or protect oocyte function, and that women with BRCAs may need special guidance in order to maintain their fertility [48].

The relationship between BRCA mutation carriers and AMH remains controversial, with a cross-sectional study showing that a lower AMH level of patients with a BRCA1 mutation than that of non-carriers [49], leaving whether AMH concentrations are associated with the BRCA2 mutation status remaining to be confirmed [49]. Similarly, Titus et al. demonstrated that young women with the BRCA1 mutation had impaired ovarian reserve by measuring serum AMH Hormone concentrations, suggesting that the DNA double-strand repair pathway was strongly associated with female oocyte aging [50]. Furthermore, a larger number of studies have shown that BRCA1 mutants have lower circulating AMH concentrations thanpatients with BRCA2 mutations of a similar age [51], and BRCA1 carriers were proved by clinical evidence to produce much less mature oocytes during ovarian stimulation than BRCA2 carriers of similar age groups [52], whereas some other studies have found no difference in serum AMH levels between BRCA carriers and non-carriers [53], of which an example is Michaelson-Cohen et al. found healthy BRCA 1/2 mutation carriers to have similar AMH level to that of women of similar age without mutations [54].

## AMH and body metabolism

According to a recent meta-analysis, women with higher vitamin D levels had a higher risk of getting pregnant after assisted reproductive technology (ART) and were more likely to have a live birth than women with lower vitamin D levels [55]. However, the relationship between the ovarian reserve and vitamin D levels is still a controversial issue on the basis of Merhi et al.’s findings that vitamin D down-regulates the expression of AMH receptor (AMHR) gene in human iuteinized granule cells by disrupting the nuclear localization of Smad 1/5/8 phosphate, indicating that in comparison with women with adequate 25OH-D (≥ 30 ng/mL), AMHR-II mRNA expression levels have doubled in women with less 25OH-D content (< 30 ng/mL) in follicular fluid, thus pointing to a negative relationship between 25OH-D levels and AMH receptor II (AMHR-II) mRNA gene expression. In addition, a study involving 388 premenopausal women conducted by Merhi et al. found that low vitamin D levels were linked to lower ovarian reserve in women of late reproductive age (≥ 40 years) [56], while on the basis of a prospective study involving 49 women of childbearing age with normal menstrual cycles, AMH levels during the first week of the menstrual cycle were raised by 12.9 ± 3.9% via providing 5000 IU of vitamin D for a week compared to the control group [57]. A study involving 283 infertile patients conducted by Drakopoulos et al. found that vitamin D levels were not associated to AMH [58], with the confirmation from Pearce et al.’s study on women with polycystic ovary syndrome (PCOS) showing no association between vitamin D levels and serum AMH [59], which was reinforced by a recent report indicating that vitamin D are not connected with AMH levels in infertile women with reduced ovarian reserve, which requires further studies to determine whether vitamin D levels affect oocyte or embryo quality, or whether hormones impact the implantation process in some ways, though [60].

### Obesity

Obesity arising among women of reproductive age can lead to serious reproductive problems like irregular menstruation, infertility, miscarriage, and poor pregnancy outcomes [61]. A cross-sectional study involving 290 infertile women conducted by Buyuk et al. suggested that high BMI was related to decreased random serum AMH levels of infertile women with reduced ovarian reserve, but not to normal ovarian function of healthy women. A comparison made between women high BMI women and those with normal BMI revealed that average random serum AMH levels were reduced by 33% in women with reduced ovarian reserve, while there is no such relation in women with normal ovarian reserve [62]. Park et al. showed that there was no remarkable association of serum AMH concentration between the normal BMI people and the obese BMI group (≥ 25 kg/m^2^) and more researches are required to determine how obesity affects AMH metabolism and clearance in various ways [63].

It is speculated that high dietary fat intake will damage women's reproductive function, thus directly affecting the morphology and function of the ovaries [64]. The mouse-based researches showed no significant differences in serum AMH concentrations between people on a low-fat and those on high-fat diet, suggesting that a high-fat diet has no effect on granulocyte function [65], while a cross-sectional study of premenopausal women found a negative relationship between dietary fat and serum AMH concentrations, thus requiring further studies to assess dietary factors as possible regulators of ovarian reserve [66].

The recovery of ovarian function in women of childbearing age with breast cancer is related to AMH and FSH levels before chemotherapy, ovarian recovery time can be estimated by the new prognostic score combined with AMH, age, and BMI, and the relevant data need to be validated in order to determine whether pre-chemotherapy ovarian reserve indicators, especially AMH, can be used to predict potential ovarian function in young patients [67].

## Guidance of AMH in choosing fertility preservation technique

AMH levels are conducive to patients and doctors choosing the right fertility maintenance methods to predict ovarian reserve after chemotherapy [68]. According to researches, about 25% of young women diagnosed with breast cancer had been engaged in inadequate fertility planning, and only a few patients had participated in fertility protection project [69]. Many young breast cancer patients are concerned that pregnancy may increase the risk of recurrence and significantly affect their survival, and that chemotherapy and radiation therapy may cause congenital disabilities, leading to the options suggested by specialists (GnRHa), such as embryo oocyte cryopreservation, immature and mature oocyte cryopreservation, ovarian tissue cryopreservation, and gonadotropin-releasing hormone agonists [69–71].

### In vitro maturation (IVM) of immature oocytes

Applied without ovarian stimulation are in vitro maturation (IVM) of immature oocytes and ovarian tissue cryopreservation (OTC), the two experimental technologies with unclear effects. The number of mature oocytes after IVM was shown in some studies to be strongly associated with serum AMH levels, with recent data showing that 8 to 20 cryopreserved oocytes after ovarian stimulation augment the possibility of live birth [72]. However, a report by controlled ovarian stimulation (COS) of infertile polycystic ovary syndrome (PCOS)women indicated that IVM-matured oocytes have a lower capacity to mature than the stimulated oocytes [73]. The optimal number of matured IVM oocytes used for fertility preservation is unlikely to be projected due to unclear capability of cancer patients’ cryopreserved IVM oocytes. As shown by the data from Sonigo et al., cryopreserving a large number of oocytes in IVM requires higher AMH and antral follicle count (AFC) levels, with at least 10 IVM oocytes requiring an AFC level greater than 20 and an AMH level greater than 3.7 ng/Ml [74].

The ovarian stimulation is required in cryopreserving embryo oocytes, both mature and immature, while serum AMH levels are inductive to assessing patient characteristics and predicting ovarian stimulation response, and can be used as an indicator of high or low response [75]. In 2013, ESHRE and NICE recommended measuring AMH before IVF to develop a personalized ovarian stimulation strategy. The NICE consensus determined that the thresholds for low and high stimulus responses are 0.75 ng/mL (5.4 pmol/L) and 3.5 ng/mL (25 pmol/L), respectively, while recently, the POSEIDON has set the AMH threshold for low stimulus response to 1.2 ng/mL (8.6 pmol/L) [76]. As shown in recent researches, personalized ovarian stimulation strategies can improve outcomes in terms of specific indicators [77], leading to the result that measuring AMH could be an essential factor in deciding the initial stimulus dose to be given [78].

For breast cancer patients without enough time to undergo ovarian stimulation, ovarian tissue harvesting and cryopreservation for eventual transplantation can be used as a fertility preservation strategy, which was supported by Oktay, K.Et al.’s report that the live birth rate of cryopreserved tissue for ovarian transplantation exceeded 30% [79], as well as a study reporting no evidence of malignant cells in cryopreserved ovarian tissue in breast cancer patients, despite the limited cancer screening methods [80]. Meanwhile, there are the researches suggesting that women over 35 should avoid ovarian tissue cryopreservation (OTC) as the method of fertility preservation due to reduced ovarian reserve and decreased oocyte quality [81]. Serum AMH levels were shown in a recent study to be the greatest indicator of primitive follicle density in a young female population with healthy ovaries, thus being considered an advisable indicator of non-growing follicular pool [82].

### Gonadotropin-releasing hormone agonist (GnRHa)

GnRH agonist inhibits ovarian function by acting on the hypothalamic-pituitary axis [83]. As for GnRHa and AMH, some studies indicated that they had no direct effect on ovarian reserve (OR), because FSH, LH, or GnRH receptors didn’t present in the original follicles [84], while some clinical trials and meta-analyses showed that premenopausal women who received GnRHa during chemotherapy had better outcomes, including lower risk of ovarian failure, higher menstrual recovery rates, and a higher probability of successful pregnancy than those who did not. However, thus leaving it a controversial issue to use GnRHa for fertility protection [85].

ASCO suggested that GnRHa should only be applied to young breast cancer patients when other options are unavailable, rather than a replacement for proven fertility preservation methods [86], which is contradicting to St. Gallen International Consensus that Ovarian function suppression (OFS) should be used during chemotherapy to treat hormone receptor negative diseases for the purpose of ovarian function and fertility preservation [87]. Luteinizing-hormone releasing-hormone analogue (LHRHa) was shown in recent randomized controlled trials to have an ovarian protective effect on HR-negative and HR-positive tumors and possibly a negative impact on the recurrence of breast cancer [88–90]. Still controversial are the possible ovarian protection mechanisms of the GnRHa, such as reducing the perfusion and chemotherapy drug delivery of the ovaries, increasing the concentration of FSH to prevent primordial follicle hyperplasia, stimulating the anti-apoptotic pathway in the ovaries, and protecting reproductive stem cells [91].

A recent research analysis on 98 premenopausal breast cancer patients receiving chemotherapy with or without GnRHa showed that the ovarian failure rate in the GnRHa group was significantly lower than in chemotherapy alone (44.7% vs. 80.6%; *p* = 0.002), and the median AMH of the GnRHa group after the first half chemotherapy cycle was significantly higher than that of the control group (1.57 ng/ml vs. 0.10 ng/ml), with the ovarian failure rates at AMH baseline level < 1.1 ng/ml of 91.3% and the baseline level > 1.1 ng/ml of 63.5% (*p* = 0.013), leading to the conclusion that the ovarian function can be assessed during chemotherapy and the ovarian failure rate can be predicted after chemotherapy due to the ability of GnRHa to protect ovarian function in young breast cancer patients who have undergone chemotherapy, whether their hormonal receptor status and AMH levels are significantly correlated with age or not, and that AMH levels can help doctors in developing fertility protection strategies during chemotherapy, as well as determining the clinical significance of GnRHa in chemotherapy [92].

In light of the fact that American Society of Clinical Oncology guidelines suggest that fertility protection should be discussed if the proposed treatment for patients of childbearing age has potential risks of infertility [93] and that embryo cryopreservation is limited by time, cost, partner or donor sperm, and the cycle of ovarian stimulation, making it difficult for many young women undergoing chemotherapy to choose assisted reproduction methods, adding GnRHa therapy to chemotherapy is more acceptable in that it can be administrated in combination with other fertility protection methods, despite the side effects such as vasomotor symptoms and reduced bone density [88].

### AMH in clinical application

AMH, a member of the TGF-β protein family in the form of NC complex consisting of N-terminal dimers and C-terminal dimers, may become a new approach to protect ovarian reserve and fertility including targeted therapy since it is generated only by the ovaries and works primarily through specific receptors expressed by the ovaries [11], with the C-terminal dimers of AMH binding to the AMHRII receptor to produce activated AMHRI and phosphorylated Smads (Smad 1/5/8) which, in combination with Smad4, enters into the nucleus to regulate AMH target genes [94]. RAMH, which is produced by recombination of the human C-terminal fragment of the AMH molecule, was found, in a recent mouse model study, able to be be transmitted to the ovary through IP injection, with the findings that that rAMH was abundant at 7 h after an injection but unavailable after 17 h, and the phosphorylation of SMAD1,5,8 began to rise at 3 h after injection, indicating that rAMH is biologically active within 3 to 6 h, but it has a relatively short half-life in vivo [95]. Since the apoptosis of the growing AMH-producing follicle granule cells occurs at 4–12 h after receiving Cy [10], injecting rAMH 30 min before Cy administration was discovered to be able to protect granulosa cells by increasing the level of AMH in the ovary. Further observation found that mice receiving rAMH combined with Cy treatment showed no significant increase in follicles compared to mice only treated with Cy after 24 h, similar proportion of follicles after 7 days and the comparable number of PMF after 3 weeks, leading to the hypothesis that rAMH might have a sustained protective effect on PMF reserve, which can be observed in protecting fertility and reproductive ability during chemotherapy. In comparison with Cy-only animals, rAMH administration maintained fertility after Cy therapy and improved the chances of pregnancy significantly, indicating that the combination treatment of rAMH in mice can protect follicle reservoirs by reducing chemotherapy-induced follicle activation and loss while maintain fertility without affecting the antitumor effects of chemotherapy [95].

It has been reported that MIS can suppress the development of breast cancer cells in vitro by interfering with cell cycle progression and inducing apoptosis by activating the NFkappaB signaling cascade, which can act as an endogenous hormone regulator of NFkappB signaling and growth in the breast [96], thus resulting in the hypotheses that AMH recombinant would be a new type of targeted new therapy in oncology and fertility protection and that AMH has the benefit of not causing systemic actions or toxicity problems since it is a natural and highly specific follicular inhibitor [97], with the molecule requiring extensive experimental researches as well as further clinical studies, though. If the results are proved to be applicable to female treatment, AMH recombinant may be a potential option for FP during chemotherapy, which requires the evaluation of the indications, the methods of administration, the timing of administration, and the potential side effects to be performed [68].

## Conclusions

Despite the fact that the breast cancer systematic treatment can bring survival benefits to patients, but it may also bring about negative impacts on fertility, especially in combination with glandular toxicity treatment, and that AMH has been, with a certain fertility-protective effect, to be the most sensitive marker for predicting ovarian function to be the most sensitive marker for predicting ovarian function, further studies are required to be conducted on both the effects of metabolism on fertility and the relationship between BRCA gene and AMH which can guide the choice of fertility protection strategies, thus showing AMH recombinant to be a novel type of targeted oncology and fertility protection therapy for young breast cancer patients.

## Data Availability

Not applicable.

## Abbreviations

AMH: Anti-müllerian hormone
MIS: Müllerian inhibiting substance
AFC: Antral follicle count
FSH: Follicle stimulating hormone
TGF-β: Transforming growth factor-beta
PMFs: Primordial follicles
Cy: Cyclophosphamide
FGF: Fibroblast growth factor
SCF: Stem cell factor
PI3K: Phosphoinositide 3-kinase
KGF: Keratinocyte growth factor
cAMP: Cyclic adenosine monophosphate
CREB: CAMP-response element-binding protein
PTEN: Phosphatase and tensin homolog
AKT: Protein kinase B
FOXO3a: Forkhead box O3a
CIA: Chemotherapy-induced amenorrhea
CRA: Chemotherapy-related amenorrhea
HER2: Human epidermalgrowth factor receptor-2
APT Trial: Adjuvant paclitaxel and trastuzumab trial
DSB: Double-strand break
BRCA: Breast Cancer Susceptibility Genes
IVF: In vitro fertilization
ART: Assisted reproductive technology
AMHR: AMH receptor
IVM: In vitro maturation
OTC: Ovarian tissue cryopreservation
PCOS: Polycystic ovary syndrome
COS: Controlled ovarian stimulation
AFC: Antral follicle count
GnRHa: Gonadotropin-releasing hormone agonist
LH: Luteinizing hormone
OR: Ovarian reserve
LHRHa: Luteinizing-hormone releasing-hormone analogue
